# Using a Real-Time Locating System to Evaluate the Impact of Telemedicine in an Emergency Department During COVID-19: Observational Study

**DOI:** 10.2196/29240

**Published:** 2021-07-26

**Authors:** Birju Patel, Stacie Vilendrer, Samantha M R Kling, Ian Brown, Ryan Ribeira, Matthew Eisenberg, Christopher Sharp

**Affiliations:** 1 Department of Medicine Stanford University School of Medicine Palo Alto, CA United States; 2 Evaluation Sciences Unit, Division of Primary Care and Population Health Department of Medicine Stanford University School of Medicine Stanford, CA United States; 3 Department of Emergency Medicine Stanford University School of Medicine Stanford, CA United States

**Keywords:** real-time locating system, wearable electronic devices, telemedicine, COVID-19, delivery of health care, real time, wearable, impact, telehealth, emergency department, observational, transmission, risk, infectious disease

## Abstract

**Background:**

Telemedicine has been deployed by health care systems in response to the COVID-19 pandemic to enable health care workers to provide remote care for both outpatients and inpatients. Although it is reasonable to suspect telemedicine visits limit unnecessary personal contact and thus decrease the risk of infection transmission, the impact of the use of such technology on clinician workflows in the emergency department is unknown.

**Objective:**

This study aimed to use a real-time locating system (RTLS) to evaluate the impact of a new telemedicine platform, which permitted clinicians located outside patient rooms to interact with patients who were under isolation precautions in the emergency department, on in-person interaction between health care workers and patients.

**Methods:**

A pre-post analysis was conducted using a badge-based RTLS platform to collect movement data including entrances and duration of stay within patient rooms of the emergency department for nursing and physician staff. Movement data was captured between March 2, 2020, the date of the first patient screened for COVID-19 in the emergency department, and April 20, 2020. A new telemedicine platform was deployed on March 29, 2020. The number of entrances and duration of in-person interactions per patient encounter, adjusted for patient length of stay, were obtained for pre- and postimplementation phases and compared with *t* tests to determine statistical significance.

**Results:**

There were 15,741 RTLS events linked to 2662 encounters for patients screened for COVID-19. There was no significant change in the number of in-person interactions between the pre- and postimplementation phases for both nurses (5.7 vs 7.0 entrances per patient, *P*=*.*07) and physicians (1.3 vs 1.5 entrances per patient, *P*=*.*12). Total duration of in-person interactions did not change (56.4 vs 55.2 minutes per patient, *P*=*.*74) despite significant increases in telemedicine videoconference frequency (0.6 vs 1.3 videoconferences per patient, *P*<.001 for change in daily average) and duration (4.3 vs 12.3 minutes per patient, *P*<.001 for change in daily average).

**Conclusions:**

Telemedicine was rapidly adopted with the intent of minimizing pathogen exposure to health care workers during the COVID-19 pandemic, yet RTLS movement data did not reveal significant changes for in-person interactions between staff and patients under investigation for COVID-19 infection. Additional research is needed to better understand how telemedicine technology may be better incorporated into emergency departments to improve workflows for frontline health care clinicians.

## Introduction

The COVID-19 pandemic acutely raised concerns about the risks of nosocomial transmission of infection within the walls of health care systems. Several health care systems experienced outbreaks [1-3], sometimes with far-reaching consequences [4]. Health care workers can become ill from nosocomial transmission [5], potentially due to patient exposure [6] and physical proximity, which cripples the ability of a health system to continue functioning at needed capacity. Personal protective equipment (PPE) has been instrumental to reduce the risk of transmission of respiratory viral illness [7,8], but given initial concerns about supply, other tools to limit exposure have been evaluated.

Telemedicine was one technology deployed by health care systems with the goal of protecting patients and staff during the COVID-19 pandemic, enabling clinicians to provide care remotely to both patients who are at home for outpatient evaluation and also on premises for acute care [9-11]. While it has been hypothesized that telemedicine would limit unnecessary in-person contact and reduce the need for PPE in health care facilities [12], the clinical and practical impact of this technology has not been validated for this purpose. For example, logs of video telecommunications can report system utilization but do not necessarily relate to risk of exposure and transmission of SARS-CoV-2 given variations in physical workflows among clinicians. Moreover, determining the impact of telemedicine on infection spread to employees is limited, as obtaining occupational health data presents legitimate privacy concerns. Direct observation of staff members after telemedicine implementation also poses safety risks during a pandemic and excludes the possibility for a baseline comparison. Yet validating the hypothesis that on-premises telemedicine reduces pathogen exposure is needed, particularly given widespread investments in these technologies for this purpose [10].

A real-time locating system (RTLS) uses a combination of sensors installed in defined locations and locators carried by staff or installed on equipment to track movement. These data may then be visible on a live monitor or recorded on a database log for further analysis, where it may provide operational or research value. For example, an RTLS can be used to measure and improve health care delivery [13] by helping understand patterns of staff movement [14-18] and monitoring operational efficiency [19,20]. The quantitative information from an RTLS can be analyzed to predict patterns in clinical workflows [21]. Recently, RTLSs have been used as a method for contact tracing during COVID-19 [22,23]. However, each RTLS deployment may have unique limitations based on its practice setting, such as partial functionality or local resistance to adoption (eg, limited staff participation with wearing tracking badges), that make its utility less clear as a broader monitoring tool [24,25].

We recognized an opportunity to evaluate whether an RTLS can quantify the impact of the rapid deployment of telemedicine on health care clinician workflows, measured by changes in movement through patient rooms. We aimed to understand (1) the feasibility of linking multimodal data, including RTLS data, to develop measures that summarize relevant physical workflows in a complex environment, and (2) describe the changes in in-person interactions between staff and patients observed after deploying a telemedicine platform in an emergency department early in the COVID-19 pandemic.

## Methods

### Implementing an RTLS in the Hospital

Stanford Health Care (SHC) launched an RTLS platform (Midmark) in conjunction with the opening of a new hospital building in late 2019. Infrared and radiofrequency sensors were installed in every patient care room, and staff were given RTLS badges to wear alongside their name badge [14,26]. The RTLS system required line of sight between the room sensor and staff member’s badge to trigger. The badge emitted a ping every 1 to 3 seconds to convey that the staff member was still present in the room, and the system only logged events lasting longer than 5 seconds. The installation team optimized sensitivity settings of the sensors based on the local geometric configuration and construction materials. Thus, if staff walked forward into a patient room wearing the locator badge clipped appropriately to their uniform, the system captured entrance and duration of time spent in the room. However, the in-room sensors did not trigger when staff members were walking down the corridor. Data were not validated by comparing logged events to visual observation by the institution or vendor except in a limited fashion during the installation process; however, prior work has found very high correlation between RTLS events and direct observation, especially when monitoring was focused on patient rooms [18]. While both nurses and physicians were encouraged to wear the badges, the technological features primarily benefitted nurses in their usual work routines: device alarms were automatically silenced by the RTLS upon entering a room, and security assistance could be summoned discreetly via an unlabeled button on the badge. Physicians who chose to wear badges could also take advantage of this security feature but were not required to participate.

### Implementing Telemedicine in the Emergency Department

At the start of the COVID-19 pandemic, SHC formed an operational committee to guide its telemedicine strategy. When the first patient was tested for COVID-19 on March 2, 2020, clinicians could activate a previously deployed communication technology (Cisco Jabber) that was primarily used for messaging between clinicians. While the technology did offer a videoconference feature, it required a staff member within the room to accept the call to initiate two-way communication. Given this limitation, a new platform composed of a consumer-grade tablet (Apple iPad) mounted on stands with wheels and a secure videoconferencing service (Zoom) was deployed in all patient care rooms throughout the emergency department [11]. To initiate a video call, clinicians activated the telemedicine system using centrally located tablets located outside of patient rooms and were automatically connected to the device at the patients’ bedside. This telemedicine system was deployed and tested by technology support staff in the adult emergency room starting March 25, 2020, and was released for use by clinicians on March 29, 2020, when clinical champions were present to demonstrate how to use the system and technology support staff were available for troubleshooting. In addition, digital resources (including video tutorials) with instructions on use were provided.

Before entering the emergency department, patients were screened for any symptoms of influenza-like illness outside of the entrance. They were then triaged into one of three pathways: low-acuity patients meeting the screening criteria for COVID-19 were sent for testing in the garage outside of the emergency department (where the RTLS was not deployed), high-acuity patients meeting the screening criteria were sent to a designated waiting room isolated from the remaining emergency department, and patients not meeting the screening criteria were sent to the primary waiting room. Once inside the emergency department, every patient who was screened for COVID-19 was placed on appropriate isolation precautions and underwent laboratory testing for SARS-CoV-2. Clinicians were able to identify patients who were being screened for COVID-19 by isolation signage on the room entrance, an indicator on the electronic locator board, and from within the electronic health record. Clinicians were free to choose whether they would interact with patients with isolation precautions in person or via telemedicine. Nonetheless, normal standards of care were expected, including physical examination of patients during the encounter.

### Developing an Analytics Pipeline and Data Analysis

We identified multiple streams of data necessary to quantify the impact of telemedicine on health care worker movement. First, we extracted the RTLS event logs for movement of nursing and physician staff in each patient care room of the emergency department. RTLS data from staff in other clinical roles, such as radiology technicians and phlebotomists, were not included in this study as these roles were not the target users of the telemedicine technology. In addition, all staff members included in the study had dedicated clinical roles in the emergency department. We also obtained utilization logs from the telemedicine platforms. Finally, we queried our electronic health record system for the isolation status of all emergency department encounters that would indicate a patient needed screening for COVID-19, along with room information, time of rooming, and time of disposition decision. Patients who were never placed in isolation for COVID-19 testing during their evaluation were excluded from the analysis, as these patients were not the target of telemedicine usage.

The RTLS and encounter data were then processed and unified to provide measurements of the primary outcomes—the number of staff entrances per qualifying emergency department encounter and the total duration spent in the patient rooms over each encounter. First, when separate, successive RTLS events linking the same staff member and room number occurred within 30 seconds of one another, these events were combined into a single event. This assumption accounts for the possibility that the line of sight between the staff member’s badge and room sensor was briefly interrupted by a shift in the location of people or equipment inside the room. We conducted sensitivity analyses using thresholds of 1 second and 10 seconds and observed no significant difference in findings. The RTLS staff movement data was then merged with data on patient encounters using room names and timestamps extracted from the electronic health record system. Since patients with longer encounters in the emergency department are naturally more likely to have more interactions with health care clinicians, measures for staff entrances and duration per encounter were adjusted by encounter length of stay (time between initial rooming and disposition time). For example, to adjust the number of entrances for each encounter, the raw number of staff entrances was divided by that encounter’s length of stay and then multiplied by the average encounter length of stay observed over the entire study period. This method of adjustment allowed for data to be analyzed at different levels of granularity, including individual encounters, daily summaries, and over the study phase. In addition, as uptake of RTLS badges was different among physicians and nurses, we generally present statistics stratified by clinical role and use rates instead of absolute counts to make interpretation more consistent.

To describe telemedicine utilization, videoconference logs from both the Cisco and Zoom systems were summarized into daily activity reports for the entire emergency department, as the technology did not track which telemedicine sessions were linked to specific patient encounters. These daily summaries were divided by the daily emergency department census of patients screened for COVID-19 (derived from encounter data) to gather an approximate number of video conferences per patient encounter.

The final data set was then separated into the pre- and postimplementation phases to understand the change over time. To allow for a similar length of observation to the preimplementation phase from March 2 to March 28, data were collected for the postimplementation phase from March 29 to April 20, 2020. For each outcome, mean (SD) values for encounter-level measures were calculated for each phase, along with *t* tests to evaluate for statistically significant differences between the two phases using an alpha of .05.

Staff and patient data were deidentified prior to being available to the analytics team. This project was not deemed human subjects research by our Institutional Review Board (protocol #55927).

## Results

### Staff and Patients

During the evaluation period, SHC cared for 6951 patients in its adult emergency department, of which 2662 (38.3%) were evaluated for COVID-19, with an average length of stay of 251 minutes. RTLS badges were worn by 245 unique staff members including 40 out of 99 (40.4%) attending physicians, 8 out of 62 (12.9%) resident physicians, and 197 (100.0%) nurses scheduled for service. Rates of badge use were similar between pre- and postimplementation phases (Table 1). We linked 15,741 RTLS events (a staff member entering a patient’s room) to the 2662 encounters of patients undergoing screening for COVID-19.

**Table 1:** Characteristics of patients, staff, and real-time locating system (RTLS) events in the emergency department stratified by study periods. Staff data report the number of staff wearing RTLS badges compared to those scheduled for at least one shift during that study phase.

Variable			Preimplementation phase		Postimplementation phase
**Patients in the emergency department**					
	All patients evaluated, n	4571		2380	
	Patients tested for COVID-19, n (%)	1502 (32.9)		1059 (44.4)	
	Length of stay for patients tested for COVID-19 (minutes), mean (SD)	190 (209)		339 (299)	
**Proportion of staff scheduled who wore an RTLS badge, n/N (%)**					
	Total staff	220/328 (67.1)		212/315 (67.3)	
	Nurses	182/182 (100.0)		174/174 (100.0)	
	Attending physicians	32/96 (33.3)		33/91 (36.3)	
	Resident physicians	6/50 (12.0)		5/50 (10.0)	
**RTLS**					
	RTLS events for COVID-19–tested patients	5855		9886	

### Uptake of Telemedicine

Staff engaged in 892 videoconferences with patients screened for COVID-19 (0.6 videoconferences per patient) during the preimplementation phase using the existing Cisco platform and 1482 videoconferences (1.3 videoconferences per patient) using either the Cisco or Zoom platform during the postimplementation phase (*P*<.001 for change in daily average of videoconferences per patient). The video functionality of the pre-existing communication platform gained use just prior to the deployment of the newer telemedicine platform, with the majority of these videoconferences (796/892, 89.2%) in the preimplementation phase occurring between March 15 and March 29. During the postimplementation phase, the newer Zoom videoconference platform was the dominant technology (906/1482 videoconferences, 61.1%). Usage of telemedicine was highest immediately after deployment (2.0 videoconferences per patient) and decreased in the subsequent weeks (1.4 videoconferences per patient in the second week after deployment and 0.9 videoconferences per patient in the third week; *P*=.002 for the difference between the first and third weeks of the postimplementation phase).

### Frequency of Patient Room Entrances

**Figure 1:**
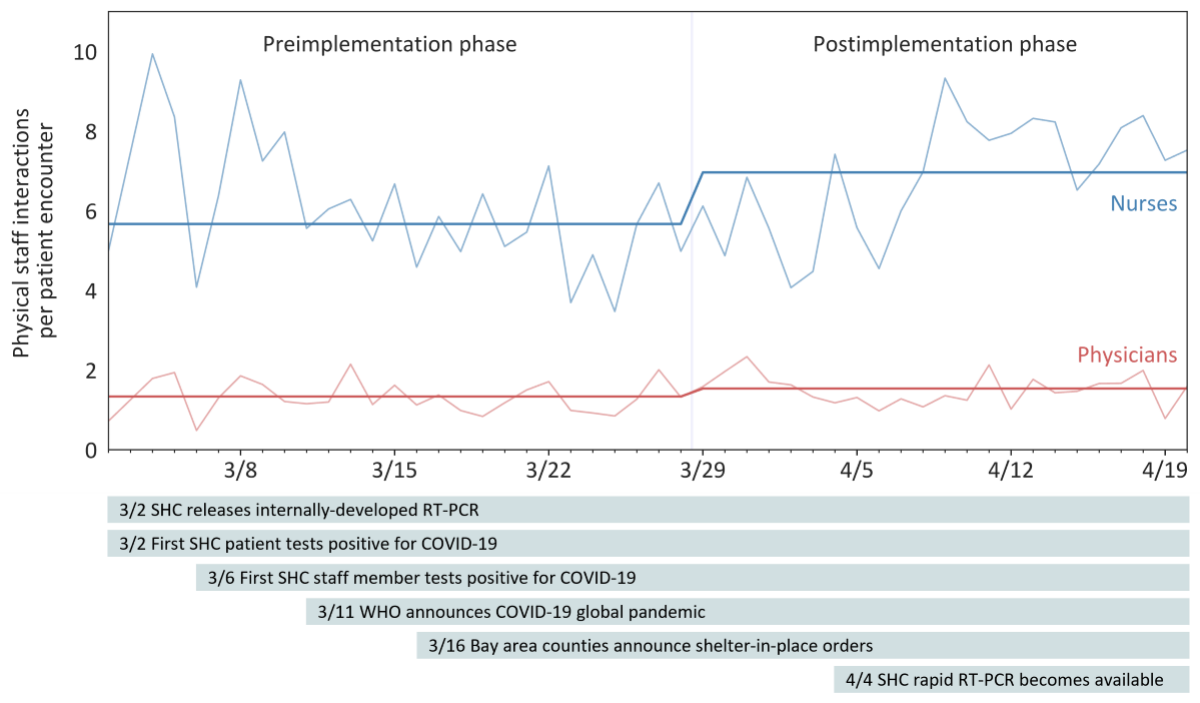
In-person staff interactions with patients under investigation for COVID-19. Entrances into rooms with patients under investigation by nurses (daily census-weighted average over phase in dark blue, daily averages in light blue) had a nonstatistically significant increase between the pre- and postimplementation phases. Physicians (daily census-weighted average over phase in dark red, daily averages in light red) physically entered patient rooms much less often than nurses, and changes in physician entrances into patient rooms over phases were more subtle in absolute counts. A timeline of relevant public health events is provided below [27-29]. SHC: Stanford Health Care, RT-PCR: reverse transcription–polymerase chain reaction, WHO: World Health Organization.

Nurses had an average of 7.3 (SD 6.9) (after adjustment for patient length of stay: mean 5.7, SD 4.1) entrances per patient encounter in the preimplementation phase. After implementation, nurses had an average of 10.8 (SD 10.2) (after adjustment: mean 7.0, SD 5.4) entrances per patient encounter, which was not a statistically significant difference between the phases (*P=.*07, Figure 1). Similarly, physicians had an average of 1.5 (SD 1.1) (after adjustment: mean 1.3, SD 1.0) entrances per patient encounter in the preimplementation phase and 2.2 (SD 2.0) (after adjustment: mean 1.5, SD 1.5) entrances per patient encounter in the postimplementation phase, which was not a statistically significant difference (*P=.*12). Further restricting the analysis to resident physicians also showed a similar pattern. In the preimplementation phase, resident physicians had an average of 1.6 (SD 1.4) (after adjustment: mean 1.1, SD 0.9) entrances per patient encounter, and 2.1 (SD 1.7) (after adjustment: mean 1.4, SD 1.0) entrances per patient encounter, which was not a statistically significant difference between the phases (*P=.*22).

### Time Spent With Patient

**Figure 2:**
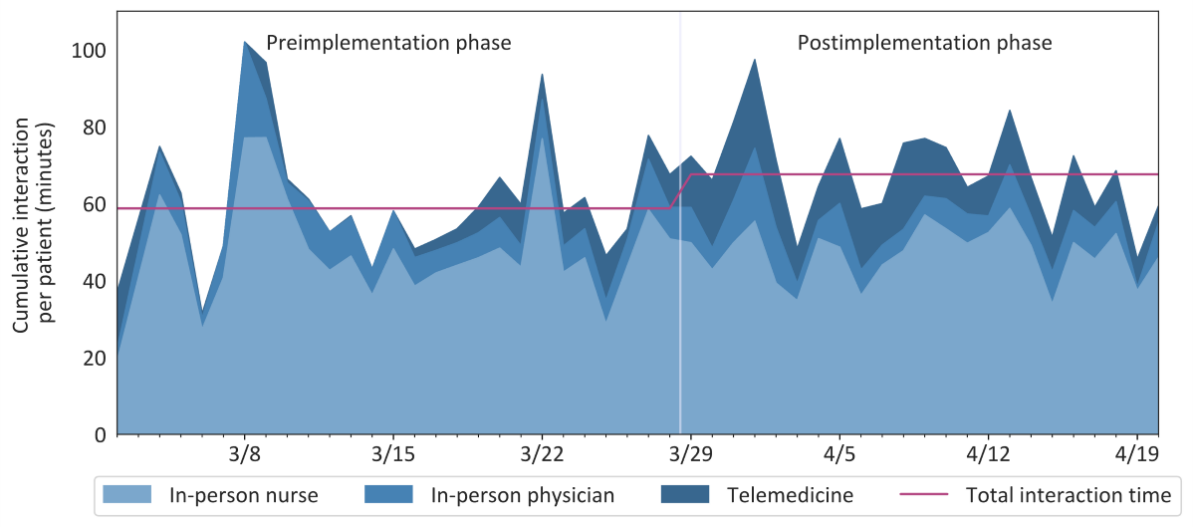
Cumulative staff-patient interaction for patients under investigation for COVID-19. In-person exposure to patients under investigation was mostly borne by nurses (daily average shaded light blue) but did not change significantly over the phases. A similar trend was seen for in-person contact time for physicians (daily average shaded blue). Virtual contact (daily average shaded dark blue) with patients increased during the postimplementation phase, demonstrating adoption of the new telemedicine technology platform beyond the previously available platform. However, there was not a statistically significant difference in total interaction time with patients between the pre- and postimplementation phases (average for entire phase shown as a purple line).

The combination of videoconferencing and RTLS data created a profile of the staff’s mode of interaction with patients. During the preimplementation phase, virtual interactions lasted an average of 4.3 minutes per encounter and accounted for 7.3% of total time staff spent with patients either in the room or via telemedicine (Figure 2). During the postimplementation phase, virtual interactions increased to 12.3 minutes per encounter and accounted for 18.2% of total time staff spent with patients (*P<.*01 for change in daily average). Simultaneously, in-person contact duration remained stable at 78.3 (SD 31.1) (after adjustment for patient length of stay: mean 56.4, SD 17.4) minutes per patient encounter during the preimplementation phase and 78.7 (SD 13.9) (after adjustment: mean 55.2, SD 8.9) minutes per patient encounter during the postimplementation phase, which was not a statistically significant difference (*P=.*74 for change in daily average). In particular, nurses spent 67.1 (SD 25.3) (after adjustment: 48.0, SD 14.3) minutes in person per patient encounter in the preimplementation phase and 67.3 (SD 11.7) (after adjustment: mean 47.2, SD 7.0) minutes in person per patient encounter in the postimplementation phase (*P*=.80), while physicians spent 11.3 (SD 7.8) (after adjustment: mean 8.5, SD 4.2) minutes in person per patient encounter in the preimplementation phase and 11.4 (SD 5.6) (after adjustment: mean 8.0, SD 3.8) minutes in person per patient encounter in the postimplementation phase (*P*=.67). Altogether, combined in-person and virtual interaction time in the postimplementation phase (mean 91.5, SD 14.4 minutes per patient encounter; after adjustment: mean 67.9, SD 11.9) was not statistically different from the preimplementation phase (mean 82.9, SD 30.1 minutes per patient encounter; after adjustment: mean 61.0, SD 17.0; *P=.*11 for change in daily average).

## Discussion

### Principal Findings

The combination of multimodal data can capture meaningful workflow components of staff movement in an emergency department during the implementation of a new software technology, even in the course of a pandemic. RTLS data have been previously utilized to evaluate clinical workflows using process mining [30] and simulation [31], and this evaluation adds an analytical approach to understand the extent that physical workflows changed after the implementation of a telemedicine technology.

With the addition of a new telemedicine platform, we expected that virtual interactions would replace a portion of in-person contact with patients and thus theoretically reduce pathogen exposure and PPE use. For example, a reduction in the number of entrances into patient care rooms may result in a reduction in PPE use. However, after cross-validating telemedicine adoption data with RTLS data, we found no significant difference in either entrances or duration of in-person physical interaction across multiple clinical roles, including nurses, attending physicians, and resident physicians, suggesting exposure risk was not reduced despite an increased use of videoconferencing. We also found that the cumulative time clinicians spent interacting with patients either in person or virtually did not change significantly in the context of a pandemic and new workflows.

Not unexpectedly and consistent with prior findings [14,32], we observed that nurses, compared to physicians, had the majority of interactions (81% of entrances and 85% of the time) with patients under evaluation for COVID-19. Thus, telemedicine technologies may hold greater potential to impact nursing workflows to decrease transmission risk in a pandemic compared to other health care clinicians; however, additional research is needed to understand what elements of nursing work can be done virtually while maintaining high-quality patient care [33]. For example, while some essential nursing tasks like medication administration need to be done in person, some aspects of communication around those actions could be made virtual. For physicians, several components of their workflows can be completed reasonably using telemedicine [34], but our data suggest that there remain important elements of clinical evaluation, communication, and treatment that physicians prioritize completing in person. Thus, telemedicine may interact with clinical workflows in more complex ways that do not manifest in our presented metrics.

In addition, other concurrent changes in technology and care standards may offer insights into why patient room entrances and time spent in person with the patient did not differ in the pre- and postimplementation of telemedicine technology. First, the development of a rapid SARS-CoV-2 RT-PCR (reverse transcription–polymerase chain reaction) assay early in the postimplementation phase may have allowed physicians to delay in-person evaluations of patients until they received test results, permitting clinicians to modify their routines depending on perceived risk after a negative COVID-19 result. Second, as emergency department staff became more comfortable with isolation protocols, they may have felt less fear of contracting the disease, making them more comfortable spending time in person with patients even when a given clinical activity, such as decision-making or counseling, could have been done via telemedicine. Lastly, during the early period of the pandemic when PPE resources were scarce, nurses may have also consolidated tasks to minimize entrances into isolation rooms. These behavioral and workflow changes may have reverted to baseline levels as the PPE inventory stabilized. However, there was a gap in the timeline between these events both before and after the telemedicine deployment, and telemedicine adoption was greatest immediately after deployment. Thus, the likelihood of observing a significant change due to the technology was high if that signal truly existed. The lack of significant differences in room entrances and total time spent with patients between the pre- and postimplementation periods may simply be a reflection of this particular technology deployment at a specific phase of the pandemic response, characterized by simultaneous and often transient complexities. Most importantly, an RTLS-based analysis can serve as a useful template for future evaluations of health technologies during emergencies, particularly if a contemporaneous comparator can be found.

### Limitations

This evaluation has certain limitations. The RTLS data and telemedicine data were unable to be merged by patient room, limiting our understanding of how staff used telemedicine and how workflow was affected for specific patient groups. Understanding the differing use of telemedicine by nurses and physicians (which was not available in our study) may be a fruitful area for future research, particularly if such data can be matched to individual staff movement data. Finally, as both attending and resident physicians did not universally wear RTLS badges, the physicians included in our analysis may not be representative of others who chose not to wear an RTLS badge. A clear strength of the evaluation, however, is that movement data was available for all nursing staff within a single emergency department and was able to be linked to individual patient encounters.

### Conclusion

Movement data captured by an RTLS is a rich technological resource that can assist in monitoring the impact of changes in workflow even in a rapidly changing clinical environment. We were able to leverage an RTLS to quantify staff movement in our emergency department as we deployed a new telemedicine platform during the COVID-19 pandemic. While telemedicine did see a significant increase in adoption in the emergency setting, it did not ultimately influence physician and nursing movement in and out of the rooms of patients who were under investigation for COVID-19. These findings underscore the need for additional formal evaluations to determine whether informatics interventions (and the significant resources directed to their deployment) are having their intended impact on health care worker safety. Such operationally relevant analyses can be enabled by unifying real-world data from multimodal platforms.

## Abbreviations

PPE: personal protective equipment
RT-PCR: reverse transcription–polymerase chain reaction
RTLS: real-time locating system
SHC: Stanford Health Care
